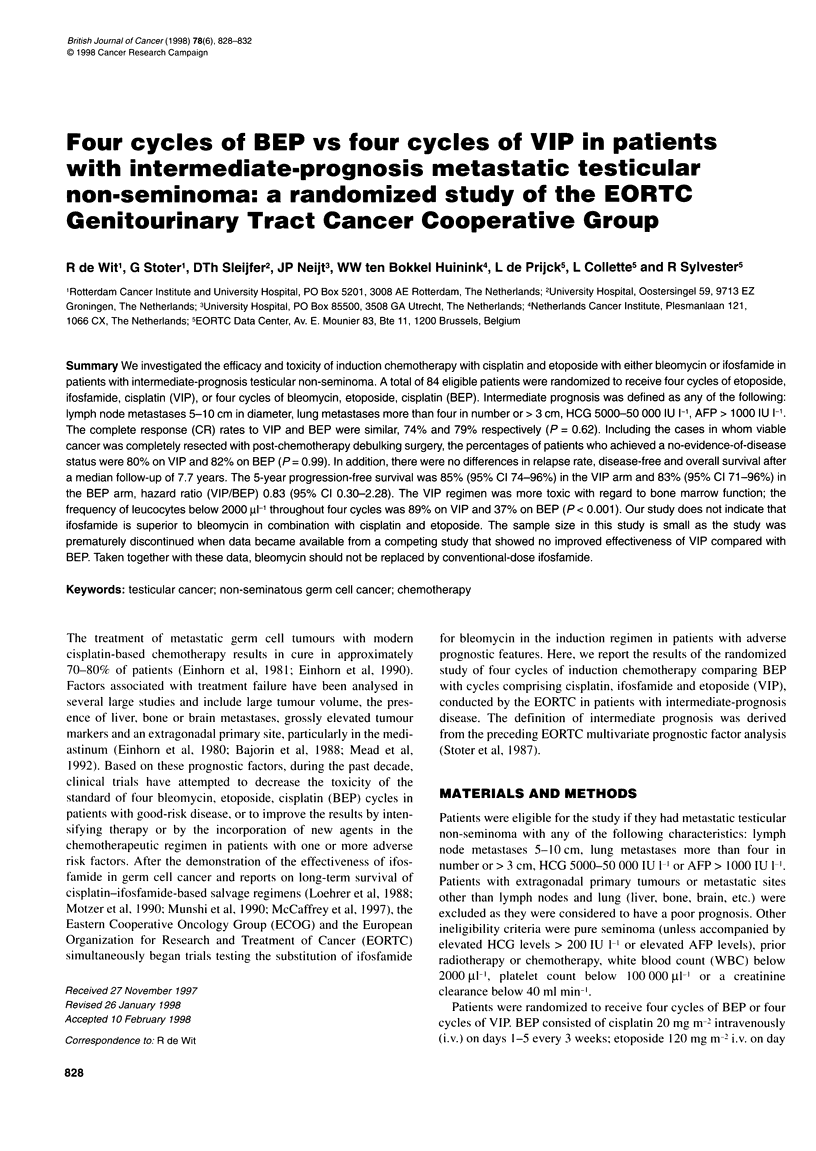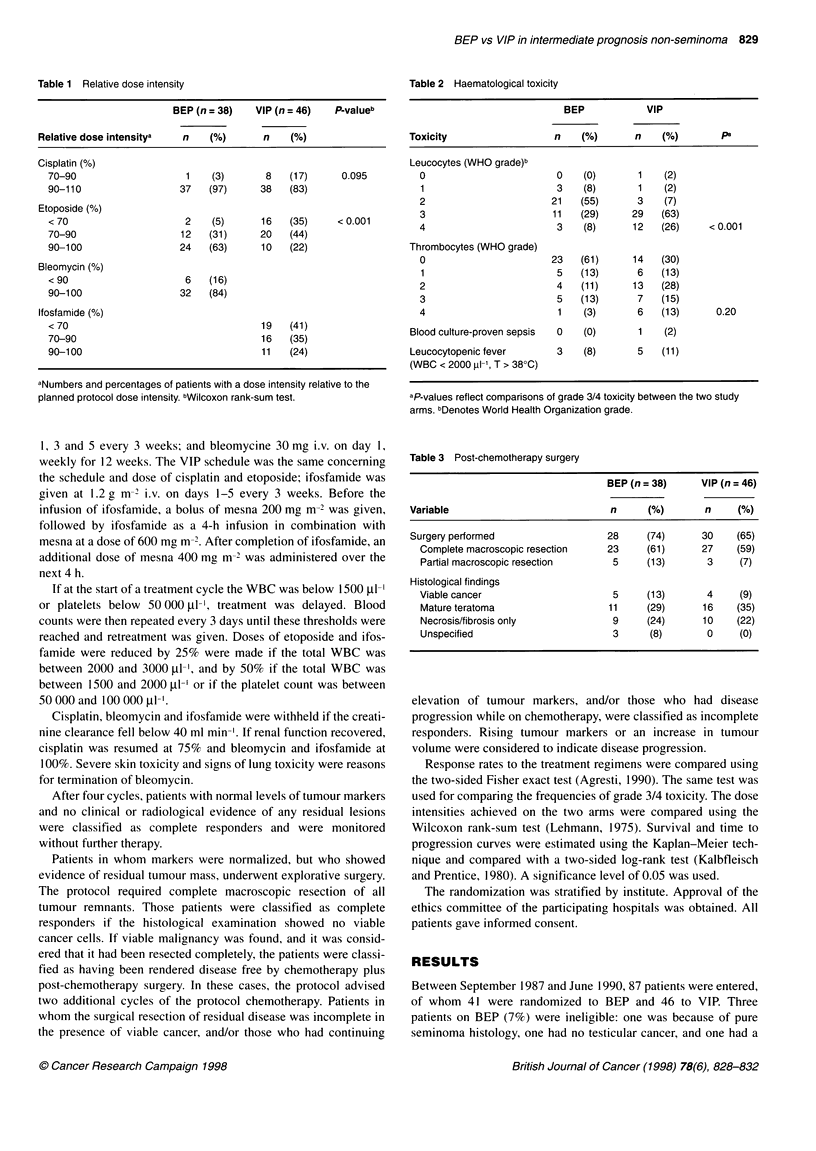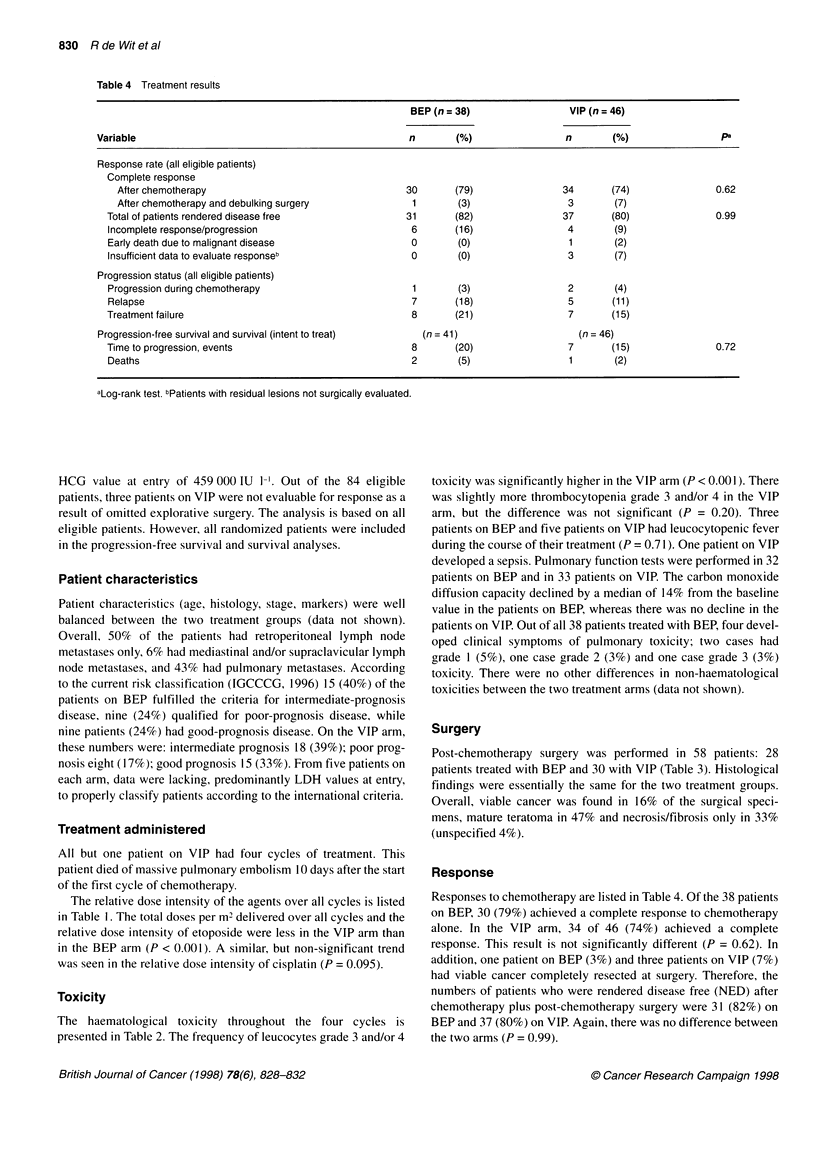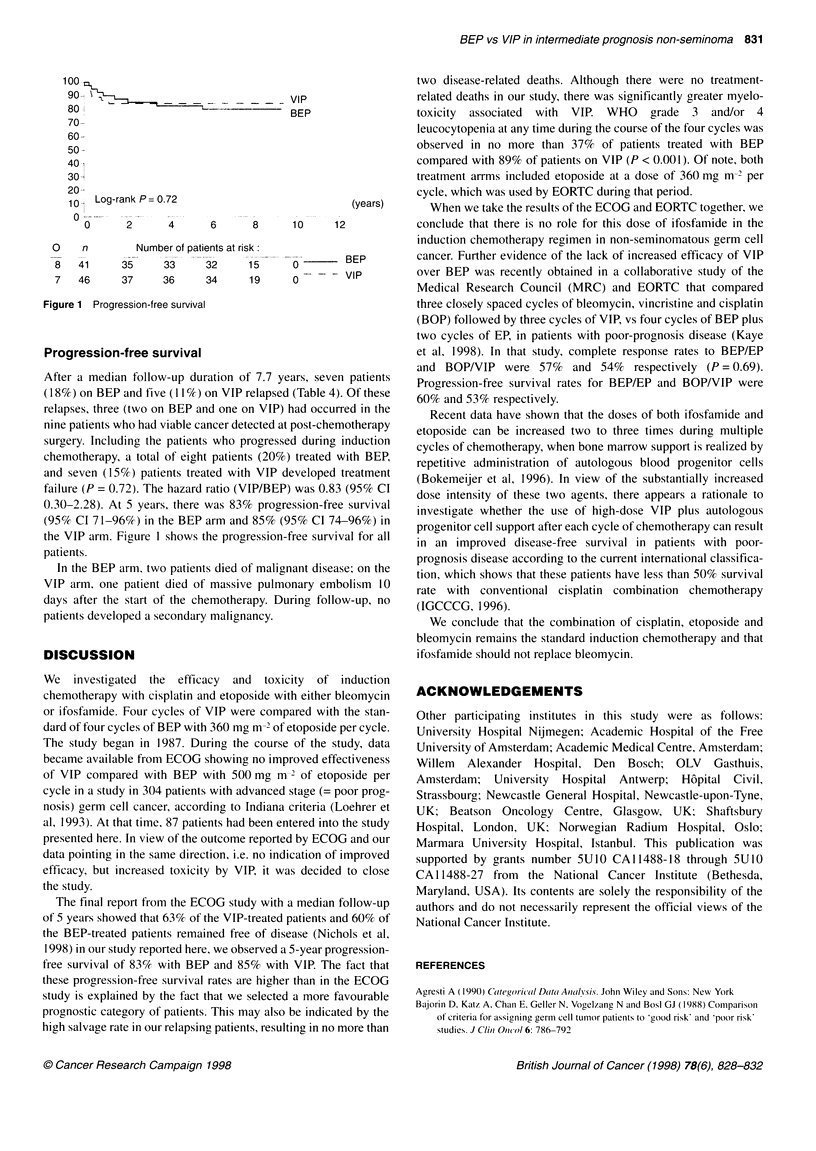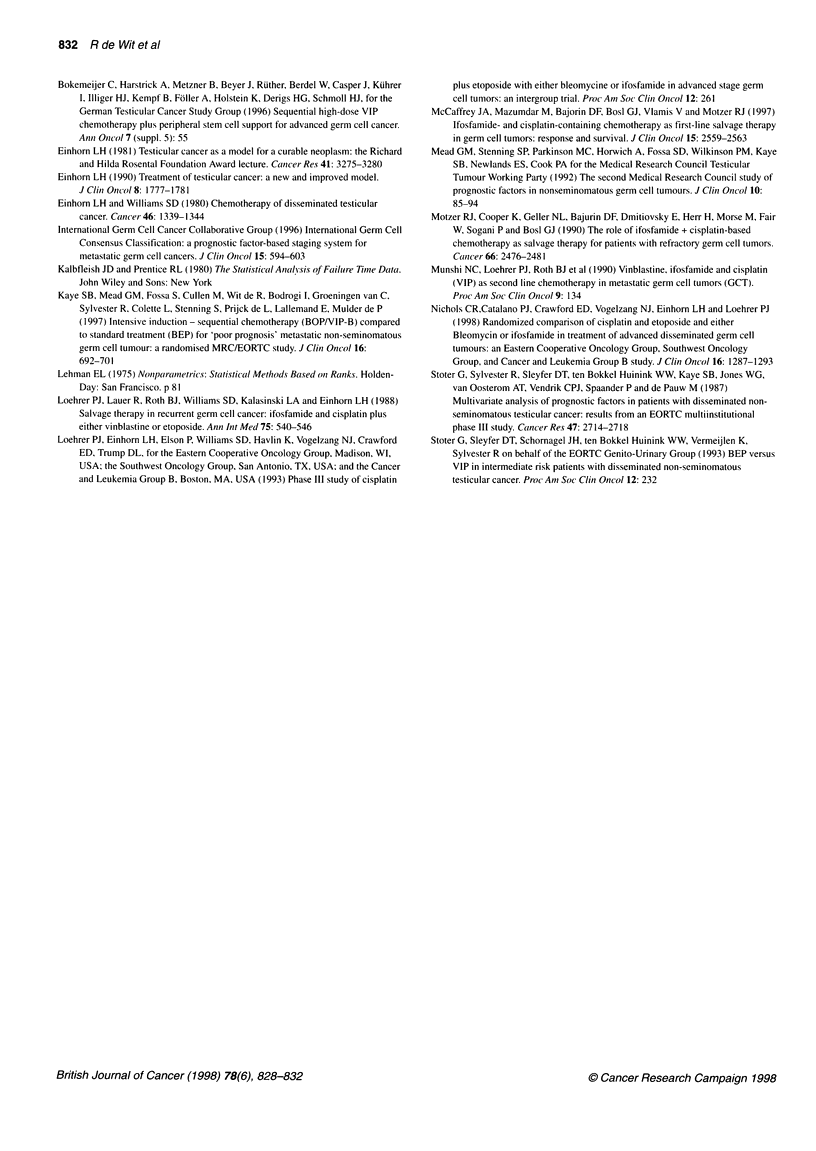# Four cycles of BEP vs four cycles of VIP in patients with intermediate-prognosis metastatic testicular non-seminoma: a randomized study of the EORTC Genitourinary Tract Cancer Cooperative Group. European Organization for Research and Treatment of Cancer.

**DOI:** 10.1038/bjc.1998.587

**Published:** 1998-09

**Authors:** R. de Wit, G. Stoter, D. T. Sleijfer, J. P. Neijt, W. W. ten Bokkel Huinink, L. de Prijck, L. Collette, R. Sylvester

**Affiliations:** Rotterdam Cancer Institute and University Hospital, The Netherlands.

## Abstract

We investigated the efficacy and toxicity of induction chemotherapy with cisplatin and etoposide with either bleomycin or ifosfamide in patients with intermediate-prognosis testicular non-seminoma. A total of 84 eligible patients were randomized to receive four cycles of etoposide, ifosfamide, cisplatin (VIP), or four cycles of bleomycin, etoposide, cisplatin (BEP). Intermediate prognosis was defined as any of the following: lymph node metastases 5-10 cm in diameter, lung metastases more than four in number or > 3 cm, HCG 5000-50,000 IU l(-1), AFP > 1000 IU l(-1). The complete response (CR) rates to VIP and BEP were similar, 74% and 79% respectively (P = 0.62). Including the cases in whom viable cancer was completely resected with post-chemotherapy debulking surgery, the percentages of patients who achieved a no-evidence-of-disease status were 80% on VIP and 82% on BEP (P = 0.99). In addition, there were no differences in relapse rate, disease-free and overall survival after a median follow-up of 7.7 years. The 5-year progression-free survival was 85% (95% CI 74-96%) in the VIP arm and 83% (95% CI 71-96%) in the BEP arm, hazard ratio (VIP/BEP) 0.83 (95% CI 0.30-2.28). The VIP regimen was more toxic with regard to bone marrow function; the frequency of leucocytes below 2000 microl(-1) throughout four cycles was 89% on VIP and 37% on BEP (P < 0.001). Our study does not indicate that ifosfamide is superior to bleomycin in combination with cisplatin and etoposide. The sample size in this study is small as the study was prematurely discontinued when data became available from a competing study that showed no improved effectiveness of VIP compared with BEP. Taken together with these data, bleomycin should not be replaced by conventional-dose ifosfamide.


British Journal of Cancer (1998) 78(6), 828–832
© 1998 Cancer Research Campaign

### Four cycles of BEP vs four cycles of VIP in patients with intermediate-prognosis metastatic testicular non-seminoma: a randomized study of the EORTC Genitourinary Tract Cancer Cooperative Group

**R de Wit[1], G Stoter[1], DTh Sleijfer[2], JP Neijt[3], WW ten Bokkel Huinink[4], L de Prijck[5], L Collette[5] and R Sylvester[5]**

[1]Rotterdam Cancer Institute and University Hospital, PO Box 5201, 3008 AE Rotterdam, The Netherlands; [2]University Hospital, Oostersingel 59, 9713 EZ Groningen, The Netherlands; [3]University Hospital, PO Box 85500, 3508 GA Utrecht, The Netherlands; [4]Netherlands Cancer Institute, Plesmanlaan 121, 1066 CX, The Netherlands; [5]EORTC Data Center, Av. E. Mounier 83, Bte 11, 1200 Brussels, Belgium

**Summary** We investigated the efficacy and toxicity of induction chemotherapy with cisplatin and etoposide with either bleomycin or ifosfamide in patients with intermediate-prognosis testicular non-seminoma. A total of 84 eligible patients were randomized to receive four cycles of etoposide, ifosfamide, cisplatin (VIP), or four cycles of bleomycin, etoposide, cisplatin (BEP). Intermediate prognosis was defined as any of the following: lymph node metastases 5–10 cm in diameter, lung metastases more than four in number or > 3 cm, HCG 5000–50 000 IU $l^{-1}$, AFP > 1000 IU $l^{-1}$. The complete response (CR) rates to VIP and BEP were similar, 74% and 79% respectively ($P = 0.62$). Including the cases in whom viable cancer was completely resected with post-chemotherapy debulking surgery, the percentages of patients who achieved a no-evidence-of-disease status were 80% on VIP and 82% on BEP ($P = 0.99$). In addition, there were no differences in relapse rate, disease-free and overall survival after a median follow-up of 7.7 years. The 5-year progression-free survival was 85% (95% CI 74–96%) in the VIP arm and 83% (95% CI 71–96%) in the BEP arm, hazard ratio (VIP/BEP) 0.83 (95% CI 0.30–2.28). The VIP regimen was more toxic with regard to bone marrow function; the frequency of leucocytes below 2000 $\mu l^{-1}$ throughout four cycles was 89% on VIP and 37% on BEP ($P < 0.001$). Our study does not indicate that ifosfamide is superior to bleomycin in combination with cisplatin and etoposide. The sample size in this study is small as the study was prematurely discontinued when data became available from a competing study that showed no improved effectiveness of VIP compared with BEP. Taken together with these data, bleomycin should not be replaced by conventional-dose ifosfamide.

**Keywords:** testicular cancer; non-seminatous germ cell cancer; chemotherapy

The treatment of metastatic germ cell tumours with modern cisplatin-based chemotherapy results in cure in approximately 70–80% of patients (Einhorn et al, 1981; Einhorn et al, 1990). Factors associated with treatment failure have been analysed in several large studies and include large tumour volume, the presence of liver, bone or brain metastases, grossly elevated tumour markers and an extragonadal primary site, particularly in the mediastinum (Einhorn et al, 1980; Bajorin et al, 1988; Mead et al, 1992). Based on these prognostic factors, during the past decade, clinical trials have attempted to decrease the toxicity of the standard of four bleomycin, etoposide, cisplatin (BEP) cycles in patients with good-risk disease, or to improve the results by intensifying therapy or by the incorporation of new agents in the chemotherapeutic regimen in patients with one or more adverse risk factors. After the demonstration of the effectiveness of ifosfamide in germ cell cancer and reports on long-term survival of cisplatin–ifosfamide-based salvage regimens (Loehrer et al, 1988; Motzer et al, 1990; Munshi et al, 1990; McCaffrey et al, 1997), the Eastern Cooperative Oncology Group (ECOG) and the European Organization for Research and Treatment of Cancer (EORTC) simultaneously began trials testing the substitution of ifosfamide for bleomycin in the induction regimen in patients with adverse prognostic features. Here, we report the results of the randomized study of four cycles of induction chemotherapy comparing BEP with cycles comprising cisplatin, ifosfamide and etoposide (VIP), conducted by the EORTC in patients with intermediate-prognosis disease. The definition of intermediate prognosis was derived from the preceding EORTC multivariate prognostic factor analysis (Stoter et al, 1987).

*Received 27 November 1997*
*Revised 26 January 1998*
*Accepted 10 February 1998*

*Correspondence to:* R de Wit

### MATERIALS AND METHODS

Patients were eligible for the study if they had metastatic testicular non-seminoma with any of the following characteristics: lymph node metastases 5–10 cm, lung metastases more than four in number or > 3 cm, HCG 5000–50 000 IU $l^{-1}$ or AFP > 1000 IU $l^{-1}$. Patients with extragonadal primary tumours or metastatic sites other than lymph nodes and lung (liver, bone, brain, etc.) were excluded as they were considered to have a poor prognosis. Other ineligibility criteria were pure seminoma (unless accompanied by elevated HCG levels > 200 IU $l^{-1}$ or elevated AFP levels), prior radiotherapy or chemotherapy, white blood count (WBC) below 2000 $\mu l^{-1}$, platelet count below 100 000 $\mu l^{-1}$ or a creatinine clearance below 40 ml $min^{-1}$.

Patients were randomized to receive four cycles of BEP or four cycles of VIP. BEP consisted of cisplatin 20 mg $m^{-2}$ intravenously (i.v.) on days 1–5 every 3 weeks; etoposide 120 mg $m^{-2}$ i.v. on day

**Table 1** Relative dose intensity

| Relative dose intensity[a] | BEP ($n$ = 38) *n* | (%) | VIP ($n$ = 46) *n* | (%) | *P*-value[b] |
|---|---|---|---|---|---|
| Cisplatin (%) | | | | | |
| 70–90 | 1 | (3) | 8 | (17) | 0.095 |
| 90–110 | 37 | (97) | 38 | (83) | |
| Etoposide (%) | | | | | |
| < 70 | 2 | (5) | 16 | (35) | < 0.001 |
| 70–90 | 12 | (31) | 20 | (44) | |
| 90–100 | 24 | (63) | 10 | (22) | |
| Bleomycin (%) | | | | | |
| < 90 | 6 | (16) | | | |
| 90–100 | 32 | (84) | | | |
| Ifosfamide (%) | | | | | |
| < 70 | | | 19 | (41) | |
| 70–90 | | | 16 | (35) | |
| 90–100 | | | 11 | (24) | |

[a]Numbers and percentages of patients with a dose intensity relative to the planned protocol dose intensity. [b]Wilcoxon rank-sum test.

**Table 2** Haematological toxicity

| Toxicity | BEP *n* | (%) | VIP *n* | (%) | *P*[a] |
|---|---|---|---|---|---|
| Leucocytes (WHO grade)[b] | | | | | |
| 0 | 0 | (0) | 1 | (2) | |
| 1 | 3 | (8) | 1 | (2) | |
| 2 | 21 | (55) | 3 | (7) | |
| 3 | 11 | (29) | 29 | (63) | |
| 4 | 3 | (8) | 12 | (26) | < 0.001 |
| Thrombocytes (WHO grade) | | | | | |
| 0 | 23 | (61) | 14 | (30) | |
| 1 | 5 | (13) | 6 | (13) | |
| 2 | 4 | (11) | 13 | (28) | |
| 3 | 5 | (13) | 7 | (15) | |
| 4 | 1 | (3) | 6 | (13) | 0.20 |
| Blood culture-proven sepsis | 0 | (0) | 1 | (2) | |
| Leucocytopenic fever (WBC < 2000 μl$^{-1}$, T > 38°C) | 3 | (8) | 5 | (11) | |

[a]*P*-values reflect comparisons of grade 3/4 toxicity between the two study arms. [b]Denotes World Health Organization grade.

1, 3 and 5 every 3 weeks; and bleomycine 30 mg i.v. on day 1, weekly for 12 weeks. The VIP schedule was the same concerning the schedule and dose of cisplatin and etoposide; ifosfamide was given at 1.2 g m$^{-2}$ i.v. on days 1–5 every 3 weeks. Before the infusion of ifosfamide, a bolus of mesna 200 mg m$^{-2}$ was given, followed by ifosfamide as a 4-h infusion in combination with mesna at a dose of 600 mg m$^{-2}$. After completion of ifosfamide, an additional dose of mesna 400 mg m$^{-2}$ was administered over the next 4 h.

If at the start of a treatment cycle the WBC was below 1500 μl$^{-1}$ or platelets below 50 000 μl$^{-1}$, treatment was delayed. Blood counts were then repeated every 3 days until these thresholds were reached and retreatment was given. Doses of etoposide and ifosfamide were reduced by 25% were made if the total WBC was between 2000 and 3000 μl$^{-1}$, and by 50% if the total WBC was between 1500 and 2000 μl$^{-1}$ or if the platelet count was between 50 000 and 100 000 μl$^{-1}$.

Cisplatin, bleomycin and ifosfamide were withheld if the creatinine clearance fell below 40 ml min$^{-1}$. If renal function recovered, cisplatin was resumed at 75% and bleomycin and ifosfamide at 100%. Severe skin toxicity and signs of lung toxicity were reasons for termination of bleomycin.

After four cycles, patients with normal levels of tumour markers and no clinical or radiological evidence of any residual lesions were classified as complete responders and were monitored without further therapy.

Patients in whom markers were normalized, but who showed evidence of residual tumour mass, underwent explorative surgery. The protocol required complete macroscopic resection of all tumour remnants. Those patients were classified as complete responders if the histological examination showed no viable cancer cells. If viable malignancy was found, and it was considered that it had been resected completely, the patients were classified as having been rendered disease free by chemotherapy plus post-chemotherapy surgery. In these cases, the protocol advised two additional cycles of the protocol chemotherapy. Patients in whom the surgical resection of residual disease was incomplete in the presence of viable cancer, and/or those who had continuing elevation of tumour markers, and/or those who had disease progression while on chemotherapy, were classified as incomplete responders. Rising tumour markers or an increase in tumour volume were considered to indicate disease progression.

**Table 3** Post-chemotherapy surgery

| Variable | BEP ($n$ = 38) *n* | (%) | VIP ($n$ = 46) *n* | (%) |
|---|---|---|---|---|
| Surgery performed | 28 | (74) | 30 | (65) |
| Complete macroscopic resection | 23 | (61) | 27 | (59) |
| Partial macroscopic resection | 5 | (13) | 3 | (7) |
| Histological findings | | | | |
| Viable cancer | 5 | (13) | 4 | (9) |
| Mature teratoma | 11 | (29) | 16 | (35) |
| Necrosis/fibrosis only | 9 | (24) | 10 | (22) |
| Unspecified | 3 | (8) | 0 | (0) |

Response rates to the treatment regimens were compared using the two-sided Fisher exact test (Agresti, 1990). The same test was used for comparing the frequencies of grade 3/4 toxicity. The dose intensities achieved on the two arms were compared using the Wilcoxon rank-sum test (Lehmann, 1975). Survival and time to progression curves were estimated using the Kaplan–Meier technique and compared with a two-sided log-rank test (Kalbfleisch and Prentice, 1980). A significance level of 0.05 was used.

The randomization was stratified by institute. Approval of the ethics committee of the participating hospitals was obtained. All patients gave informed consent.

### RESULTS

Between September 1987 and June 1990, 87 patients were entered, of whom 41 were randomized to BEP and 46 to VIP. Three patients on BEP (7%) were ineligible: one was because of pure seminoma histology, one had no testicular cancer, and one had a

© Cancer Research Campaign 1998

Table 4 Treatment results

| Variable | BEP ($n$ = 38) *n* | BEP (%) | VIP ($n$ = 46) *n* | VIP (%) | $P^a$ |
|---|---|---|---|---|---|
| Response rate (all eligible patients) | | | | | |
| Complete response | | | | | |
| After chemotherapy | 30 | (79) | 34 | (74) | 0.62 |
| After chemotherapy and debulking surgery | 1 | (3) | 3 | (7) | |
| Total of patients rendered disease free | 31 | (82) | 37 | (80) | 0.99 |
| Incomplete response/progression | 6 | (16) | 4 | (9) | |
| Early death due to malignant disease | 0 | (0) | 1 | (2) | |
| Insufficient data to evaluate response[b] | 0 | (0) | 3 | (7) | |
| Progression status (all eligible patients) | | | | | |
| Progression during chemotherapy | 1 | (3) | 2 | (4) | |
| Relapse | 7 | (18) | 5 | (11) | |
| Treatment failure | 8 | (21) | 7 | (15) | |
| Progression-free survival and survival (intent to treat) | ($n$ = 41) | | ($n$ = 46) | | |
| Time to progression, events | 8 | (20) | 7 | (15) | 0.72 |
| Deaths | 2 | (5) | 1 | (2) | |

[a]Log-rank test. [b]Patients with residual lesions not surgically evaluated.

HCG value at entry of 459 000 IU $l^{-1}$. Out of the 84 eligible patients, three patients on VIP were not evaluable for response as a result of omitted explorative surgery. The analysis is based on all eligible patients. However, all randomized patients were included in the progression-free survival and survival analyses.

### Patient characteristics

Patient characteristics (age, histology, stage, markers) were well balanced between the two treatment groups (data not shown). Overall, 50% of the patients had retroperitoneal lymph node metastases only, 6% had mediastinal and/or supraclavicular lymph node metastases, and 43% had pulmonary metastases. According to the current risk classification (IGCCCG, 1996) 15 (40%) of the patients on BEP fulfilled the criteria for intermediate-prognosis disease, nine (24%) qualified for poor-prognosis disease, while nine patients (24%) had good-prognosis disease. On the VIP arm, these numbers were: intermediate prognosis 18 (39%); poor prognosis eight (17%); good prognosis 15 (33%). From five patients on each arm, data were lacking, predominantly LDH values at entry, to properly classify patients according to the international criteria.

### Treatment administered

All but one patient on VIP had four cycles of treatment. This patient died of massive pulmonary embolism 10 days after the start of the first cycle of chemotherapy.

The relative dose intensity of the agents over all cycles is listed in Table 1. The total doses per $m^2$ delivered over all cycles and the relative dose intensity of etoposide were less in the VIP arm than in the BEP arm ($P$ < 0.001). A similar, but non-significant trend was seen in the relative dose intensity of cisplatin ($P$ = 0.095).

### Toxicity

The haematological toxicity throughout the four cycles is presented in Table 2. The frequency of leucocytes grade 3 and/or 4 toxicity was significantly higher in the VIP arm ($P$ < 0.001). There was slightly more thrombocytopenia grade 3 and/or 4 in the VIP arm, but the difference was not significant ($P$ = 0.20). Three patients on BEP and five patients on VIP had leucocytopenic fever during the course of their treatment ($P$ = 0.71). One patient on VIP developed a sepsis. Pulmonary function tests were performed in 32 patients on BEP and in 33 patients on VIP. The carbon monoxide diffusion capacity declined by a median of 14% from the baseline value in the patients on BEP, whereas there was no decline in the patients on VIP. Out of all 38 patients treated with BEP, four developed clinical symptoms of pulmonary toxicity; two cases had grade 1 (5%), one case grade 2 (3%) and one case grade 3 (3%) toxicity. There were no other differences in non-haematological toxicities between the two treatment arms (data not shown).

### Surgery

Post-chemotherapy surgery was performed in 58 patients: 28 patients treated with BEP and 30 with VIP (Table 3). Histological findings were essentially the same for the two treatment groups. Overall, viable cancer was found in 16% of the surgical specimens, mature teratoma in 47% and necrosis/fibrosis only in 33% (unspecified 4%).

### Response

Responses to chemotherapy are listed in Table 4. Of the 38 patients on BEP, 30 (79%) achieved a complete response to chemotherapy alone. In the VIP arm, 34 of 46 (74%) achieved a complete response. This result is not significantly different ($P$ = 0.62). In addition, one patient on BEP (3%) and three patients on VIP (7%) had viable cancer completely resected at surgery. Therefore, the numbers of patients who were rendered disease free (NED) after chemotherapy plus post-chemotherapy surgery were 31 (82%) on BEP and 37 (80%) on VIP. Again, there was no difference between the two arms ($P$ = 0.99).

© Cancer Research Campaign 1998

**Figure 1** Progression-free survival

### Progression-free survival

After a median follow-up duration of 7.7 years, seven patients (18%) on BEP and five (11%) on VIP relapsed (Table 4). Of these relapses, three (two on BEP and one on VIP) had occurred in the nine patients who had viable cancer detected at post-chemotherapy surgery. Including the patients who progressed during induction chemotherapy, a total of eight patients (20%) treated with BEP, and seven (15%) patients treated with VIP developed treatment failure ($P = 0.72$). The hazard ratio (VIP/BEP) was 0.83 (95% CI 0.30–2.28). At 5 years, there was 83% progression-free survival (95% CI 71–96%) in the BEP arm and 85% (95% CI 74–96%) in the VIP arm. Figure 1 shows the progression-free survival for all patients.

In the BEP arm, two patients died of malignant disease; on the VIP arm, one patient died of massive pulmonary embolism 10 days after the start of the chemotherapy. During follow-up, no patients developed a secondary malignancy.

### DISCUSSION

We investigated the efficacy and toxicity of induction chemotherapy with cisplatin and etoposide with either bleomycin or ifosfamide. Four cycles of VIP were compared with the standard of four cycles of BEP with 360 mg $m^{-2}$ of etoposide per cycle. The study began in 1987. During the course of the study, data became available from ECOG showing no improved effectiveness of VIP compared with BEP with 500 mg $m^{-2}$ of etoposide per cycle in a study in 304 patients with advanced stage (= poor prognosis) germ cell cancer, according to Indiana criteria (Loehrer et al, 1993). At that time, 87 patients had been entered into the study presented here. In view of the outcome reported by ECOG and our data pointing in the same direction, i.e. no indication of improved efficacy, but increased toxicity by VIP, it was decided to close the study.

The final report from the ECOG study with a median follow-up of 5 years showed that 63% of the VIP-treated patients and 60% of the BEP-treated patients remained free of disease (Nichols et al, 1998) in our study reported here, we observed a 5-year progression-free survival of 83% with BEP and 85% with VIP. The fact that these progression-free survival rates are higher than in the ECOG study is explained by the fact that we selected a more favourable prognostic category of patients. This may also be indicated by the high salvage rate in our relapsing patients, resulting in no more than two disease-related deaths. Although there were no treatment-related deaths in our study, there was significantly greater myelotoxicity associated with VIP. WHO grade 3 and/or 4 leucocytopenia at any time during the course of the four cycles was observed in no more than 37% of patients treated with BEP compared with 89% of patients on VIP ($P < 0.001$). Of note, both treatment arrms included etoposide at a dose of 360 mg $m^{-2}$ per cycle, which was used by EORTC during that period.

When we take the results of the ECOG and EORTC together, we conclude that there is no role for this dose of ifosfamide in the induction chemotherapy regimen in non-seminomatous germ cell cancer. Further evidence of the lack of increased efficacy of VIP over BEP was recently obtained in a collaborative study of the Medical Research Council (MRC) and EORTC that compared three closely spaced cycles of bleomycin, vincristine and cisplatin (BOP) followed by three cycles of VIP, vs four cycles of BEP plus two cycles of EP, in patients with poor-prognosis disease (Kaye et al, 1998). In that study, complete response rates to BEP/EP and BOP/VIP were 57% and 54% respectively ($P = 0.69$). Progression-free survival rates for BEP/EP and BOP/VIP were 60% and 53% respectively.

Recent data have shown that the doses of both ifosfamide and etoposide can be increased two to three times during multiple cycles of chemotherapy, when bone marrow support is realized by repetitive administration of autologous blood progenitor cells (Bokemeijer et al, 1996). In view of the substantially increased dose intensity of these two agents, there appears a rationale to investigate whether the use of high-dose VIP plus autologous progenitor cell support after each cycle of chemotherapy can result in an improved disease-free survival in patients with poor-prognosis disease according to the current international classification, which shows that these patients have less than 50% survival rate with conventional cisplatin combination chemotherapy (IGCCCG, 1996).

We conclude that the combination of cisplatin, etoposide and bleomycin remains the standard induction chemotherapy and that ifosfamide should not replace bleomycin.

### ACKNOWLEDGEMENTS

Other participating institutes in this study were as follows: University Hospital Nijmegen; Academic Hospital of the Free University of Amsterdam; Academic Medical Centre, Amsterdam; Willem Alexander Hospital, Den Bosch; OLV Gasthuis, Amsterdam; University Hospital Antwerp; Hôpital Civil, Strassbourg; Newcastle General Hospital, Newcastle-upon-Tyne, UK; Beatson Oncology Centre, Glasgow, UK; Shaftsbury Hospital, London, UK; Norwegian Radium Hospital, Oslo; Marmara University Hospital, Istanbul. This publication was supported by grants number 5U10 CA11488-18 through 5U10 CA11488-27 from the National Cancer Institute (Bethesda, Maryland, USA). Its contents are solely the responsibility of the authors and do not necessarily represent the official views of the National Cancer Institute.

### REFERENCES

Agresti A (1990) *Categorical Data Analysis*. John Wiley and Sons: New York

Bajorin D, Katz A, Chan E, Geller N, Vogelzang N and Bosl GJ (1988) Comparison of criteria for assigning germ cell tumor patients to 'good risk' and 'poor risk' studies. *J Clin Oncol* **6**: 786–792

© Cancer Research Campaign 1998

Bokemeijer C, Harstrick A, Metzner B, Beyer J, Rüther, Berdel W, Casper J, Kührer I, Illiger HJ, Kempf B, Föller A, Holstein K, Derigs HG, Schmoll HJ, for the German Testicular Cancer Study Group (1996) Sequential high-dose VIP chemotherapy plus peripheral stem cell support for advanced germ cell cancer. *Ann Oncol* **7** (suppl. 5): 55

Einhorn LH (1981) Testicular cancer as a model for a curable neoplasm: the Richard and Hilda Rosental Foundation Award lecture. *Cancer Res* **41**: 3275–3280

Einhorn LH (1990) Treatment of testicular cancer: a new and improved model. *J Clin Oncol* **8**: 1777–1781

Einhorn LH and Williams SD (1980) Chemotherapy of disseminated testicular cancer. *Cancer* **46**: 1339–1344

International Germ Cell Cancer Collaborative Group (1996) International Germ Cell Consensus Classification: a prognostic factor-based staging system for metastatic germ cell cancers. *J Clin Oncol* **15**: 594–603

Kalbfleish JD and Prentice RL (1980) *The Statistical Analysis of Failure Time Data*. John Wiley and Sons: New York

Kaye SB, Mead GM, Fossa S, Cullen M, Wit de R, Bodrogi I, Groeningen van C, Sylvester R, Colette L, Stenning S, Prijck de L, Lallemand E, Mulder de P (1997) Intensive induction – sequential chemotherapy (BOP/VIP-B) compared to standard treatment (BEP) for 'poor prognosis' metastatic non-seminomatous germ cell tumour: a randomised MRC/EORTC study. *J Clin Oncol* **16**: 692–701

Lehman EL (1975) *Nonparametrics: Statistical Methods Based on Ranks*. Holden-Day: San Francisco. p 81

Loehrer PJ, Lauer R, Roth BJ, Williams SD, Kalasinski LA and Einhorn LH (1988) Salvage therapy in recurrent germ cell cancer: ifosfamide and cisplatin plus either vinblastine or etoposide. *Ann Int Med* **75**: 540–546

Loehrer PJ, Einhorn LH, Elson P, Williams SD, Havlin K, Vogelzang NJ, Crawford ED, Trump DL, for the Eastern Cooperative Oncology Group, Madison, WI, USA; the Southwest Oncology Group, San Antonio, TX, USA; and the Cancer and Leukemia Group B, Boston, MA, USA (1993) Phase III study of cisplatin plus etoposide with either bleomycine or ifosfamide in advanced stage germ cell tumors: an intergroup trial. *Proc Am Soc Clin Oncol* **12**: 261

McCaffrey JA, Mazumdar M, Bajorin DF, Bosl GJ, Vlamis V and Motzer RJ (1997) Ifosfamide- and cisplatin-containing chemotherapy as first-line salvage therapy in germ cell tumors: response and survival. *J Clin Oncol* **15**: 2559–2563

Mead GM, Stenning SP, Parkinson MC, Horwich A, Fossa SD, Wilkinson PM, Kaye SB, Newlands ES, Cook PA for the Medical Research Council Testicular Tumour Working Party (1992) The second Medical Research Council study of prognostic factors in nonseminomatous germ cell tumours. *J Clin Oncol* **10**: 85–94

Motzer RJ, Cooper K, Geller NL, Bajurin DF, Dmitiovsky E, Herr H, Morse M, Fair W, Sogani P and Bosl GJ (1990) The role of ifosfamide + cisplatin-based chemotherapy as salvage therapy for patients with refractory germ cell tumors. *Cancer* **66**: 2476–2481

Munshi NC, Loehrer PJ, Roth BJ et al (1990) Vinblastine, ifosfamide and cisplatin (VIP) as second line chemotherapy in metastatic germ cell tumors (GCT). *Proc Am Soc Clin Oncol* **9**: 134

Nichols CR,Catalano PJ, Crawford ED, Vogelzang NJ, Einhorn LH and Loehrer PJ (1998) Randomized comparison of cisplatin and etoposide and either Bleomycin or ifosfamide in treatment of advanced disseminated germ cell tumours: an Eastern Cooperative Oncology Group, Southwest Oncology Group, and Cancer and Leukemia Group B study. *J Clin Oncol* **16**: 1287–1293

Stoter G, Sylvester R, Sleyfer DT, ten Bokkel Huinink WW, Kaye SB, Jones WG, van Oosterom AT, Vendrik CPJ, Spaander P and de Pauw M (1987) Multivariate analysis of prognostic factors in patients with disseminated non-seminomatous testicular cancer: results from an EORTC multiinstitutional phase III study. *Cancer Res* **47**: 2714–2718

Stoter G, Sleyfer DT, Schornagel JH, ten Bokkel Huinink WW, Vermeijlen K, Sylvester R on behalf of the EORTC Genito-Urinary Group (1993) BEP versus VIP in intermediate risk patients with disseminated non-seminomatous testicular cancer. *Proc Am Soc Clin Oncol* **12**: 232

© Cancer Research Campaign 1998